# The Comparative Value of Serum Angiotensin Converting Enzyme (ACE) and Lysozyme and the Use of Polyclonal Antibody Activation in the Work-up of Ocular Sarcoidosis

**DOI:** 10.3390/diagnostics11040608

**Published:** 2021-03-29

**Authors:** Ioannis Papasavvas, Béatrice Gehrig, Carl P. Herbort

**Affiliations:** Retinal and Inflammatory Eye Diseases, Centre for Ophthalmic Specialized Care (COS), 1003 Lausanne, Switzerland; i.s.papasavvas@gmail.com (I.P.); beabrunocos@gmail.com (B.G.)

**Keywords:** angiotensin converting enzyme (ACE), lysozyme, polyclonal antibody activation, ocular sarcoidosis

## Abstract

Background: The diagnosis of ocular sarcoidosis (OS) is difficult to establish in the absence of manifest systemic involvement. To help clinicians reach a diagnosis, we convened a group of experts in 2006 (International Workshop on Ocular Sarcoidosis (IWOS)) to set-up clinical criteria for the diagnosis of ocular sarcoidosis. In addition, laboratory investigational tests represent a much-needed adjunct to ascertain the diagnosis. However, many of these tests have low sensitivity and specificity. Purpose: The aim of our study was to evaluate the usefulness of serum ACE, serum lysozyme and polyclonal antibody activation in the diagnosis of ocular sarcoidosis and compare the frequency of increased serum levels of lysozyme and ACE in proven ocular sarcoidosis or in suspected ocular sarcoidosis. Methods: Serum ACE and lysozyme were assessed in these two groups and their means compared to a group of non-granulomatous (i.e., non-sarcoidosis) uveitis patients. The proportion of elevated serum ACE versus lysozyme was compared in the sarcoidosis patients. Polyclonal antibody activation was measured by establishing exposition of patients to four human commensal herpesviruses (EBV, CMV, HSV and VZV) using ELISA or immunofluorescence and in parallel by performing quantitative complement fixation (CF) serologies. The ratio of elevated CF to positive ELISA/immunofluorescence serologies was calculated. The mean of ratios (polyclonal antibody activation) was compared between ocular sarcoidosis and control groups. Results: Thirty-seven patients (F24/M13) were included in our study including 17 patients with IWOS Level 1 and 2 criteria qualifying for Group 1 (proven sarcoidosis) and 20 ocular sarcoidosis suspect patients. Mean age was 54.52 ± 23.74. Mean serum levels of ACE was 49.17± 29 IU/L in the ocular sarcoidosis group versus 27.4 ± 15.34 IU/L (*p* ≤ 0.00018, student’s t test) in the control group. Mean serum lysozyme levels was 39.92 ± 55.5 mg/L in the ocular sarcoidosis group versus 10.5 ± 5.8 mg/L (*p* ≤ 0.0013) in the control group (*n* = 30). Both tests were elevated in 8/37 (21.6%) patients, elevated ACE and normal lysozyme was noted in 2/37 (5.4%) patients, whereas the proportion of normal ACE/elevated lysozyme was much higher, 23/37 (62.2%). In 4/37 (10.8%) patients, both tests were normal. The mean score of polyclonal activation (N of elevated CF serologies divided by number of viruses to which a patient was exposed) was 0.6 ± 0.33 in the ocular sarcoidosis group versus 0.15 ± 0.2 for the control group (*n* = 42) (*p* ≤ 0.00001). Sensitivity and specificity of ACE and lysozyme were, respectively, 27%/96.6% and 83.7%/90%. Sensitivity and specificity of polyclonal antibody activation amounted to 70%/90.4% Conclusion: Lysozyme was found to be much more useful than ACE as a laboratory test to support the diagnosis of ocular sarcoidosis. As shown in a previous study, polyclonal antibody activation appears to be another useful laboratory test supportive of the diagnosis of ocular sarcoidosis.

## 1. Introduction

The diagnosis of ocular sarcoidosis is difficult to establish. It was the aim of the organization of the International Workshops on Ocular Sarcoidosis (IWOS) to set diagnostic criteria in order to facilitate the diagnosis of ocular sarcoidosis. The first IWOS was held in Tokyo in 2006 and resulted in a set of clinical criteria for the diagnosis of ocular sarcoidosis [1]. Despite these guidelines, it is often difficult to pose the diagnosis with a high degree of certainty. Laboratory tests are helpful but mostly have unsatisfactory levels of specificity and sensitivity. Therefore, multiple tests are performed to increase the probability of the diagnosis. Several articles report an increase of immunoglobulins or polyclonal antibody activation in systemic sarcoidosis [2,3,4,5,6,7]. We also found polyclonal antibody rise or activation in patients with ocular sarcoidosis and suggested to use this feature as a complimentary test for ocular sarcoidosis [8]. Among often performed laboratory tests, serum angiotensin converting enzyme (ACE) is classically recommended [9,10,11]. In our daily practice, serum ACE dosage is often normal and not helpful for diagnosis. Moreover, this test is affected by ACE inhibitors used as anti-hypertensive treatment. On the other hand, serum lysozyme seems more often elevated compared to ACE in our ocular sarcoidosis cases. Lysozyme, similar to ACE, is produced by granulomas and is shed into the blood [12,13]. However, lysozyme is rarely recommended as a complimentary test for ocular sarcoidosis and is rarely reported in studies. Even in their exhaustive review on ocular sarcoidosis, Varon et al. neglected the role of lysozyme in the work-up of OS [14]. It was this observation in our daily practice that prompted us to perform this study as well as to find new easily performed tests that can lead to a more certain diagnosis of OS. The aim was to evaluate the usefulness of serum ACE, serum lysozyme and polyclonal antibody activation and compare the frequency of increased serum levels of lysozyme and ACE and their sensitivities and specificities in proven ocular sarcoidosis or in suspected ocular sarcoidosis cases. The diagnosis was based on the IWOS clinical diagnosis criteria. [1]. In parallel, we investigated and aimed at reconfirming the utility of polyclonal antibody activation reported in a previous series [8].

## 2. Methods

### 2.1. Inclusion Criteria

We included patients with ocular sarcoidosis according to the modified IWOS criteria in a retrospective fashion. Patients were divided into two categories: (1) proven ocular sarcoidosis patients; and (2) ocular sarcoidosis suspect patients. The first category of patients corresponded to Levels 1 and 2 of the IWOS criteria, i.e., biopsy proven sarcoidosis patients with a compatible uveitis (IWOS Level 1) or patients with a compatible uveitis, where the chest X-ray or CT scan revealed the presence of bilateral hilar lymphadenopathy (BHL) and/or parenchymal peri-lymphatic micronodular pattern (IWOS Level 2).

**Figure 1:**
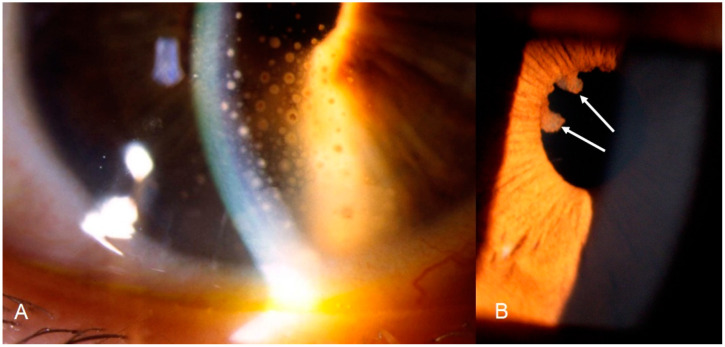
Ocular sarcoidosis, clinical signs: (**A**) granulomatous (mutton-fat) keratic precipitates on the inner surface of the cornea (KPs); and (**B**) Koeppe nodules (arrows) at the margin of the pupil.

**Figure 2:**
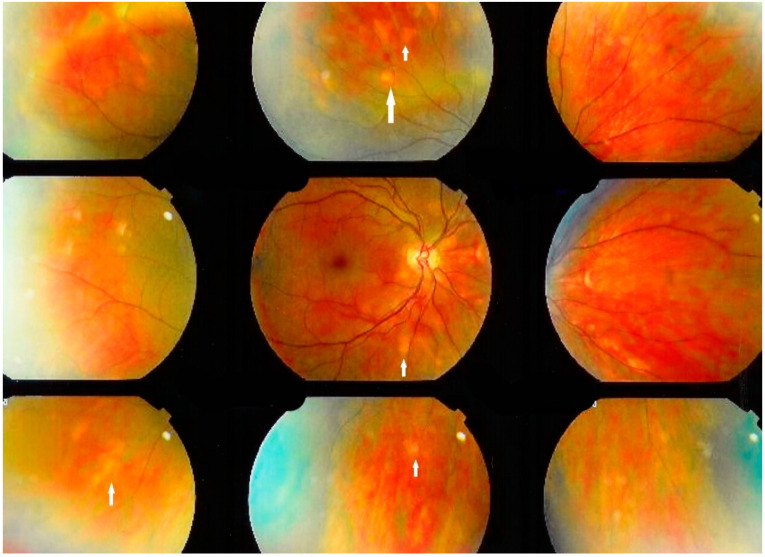
Ocular sarcoidosis, clinical signs: Numerous fundus granulomas (white arrows) appearing as disseminated yellow depigmented areas.

**Figure 3:**
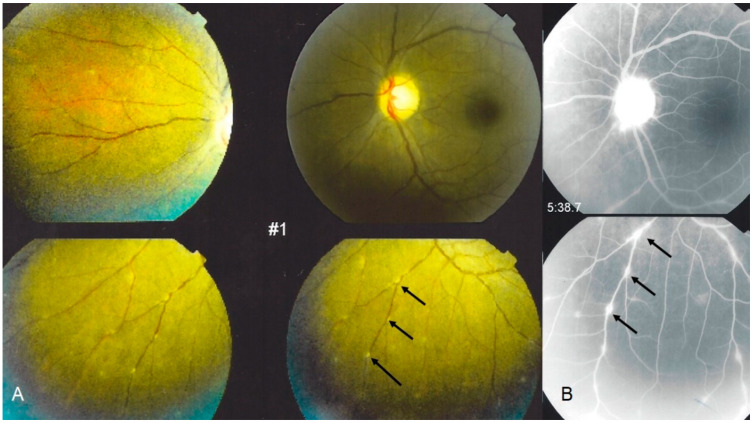
Ocular sarcoidosis, clinical signs: typical candle-wax dripping vasculitis shown on: (**A**) fundus pictures (arrows), corresponding to leaking vessels; and (**B**) fluorescein angiography (arrows). Note also a hyperfluorescent inflamed optic disc ((**B**) top photo).

**Figure 4:**
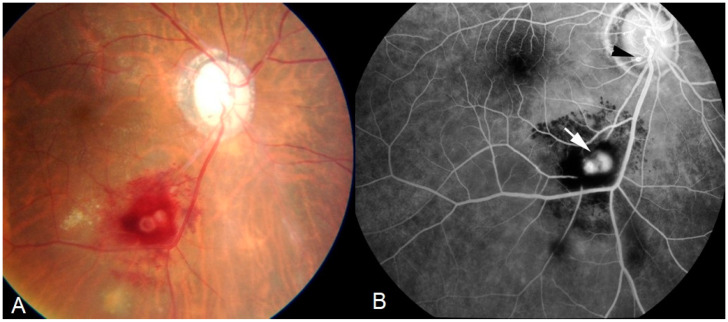
Ocular sarcoidosis, clinical signs: retinal macro-aneurism (white arrow) is a typical finding. Small microaneurysm at optic nerf head (black arrowhead). Aspect on: fundus photography (**A**); and fluorescein angiography (**B**).

**Figure 5:**
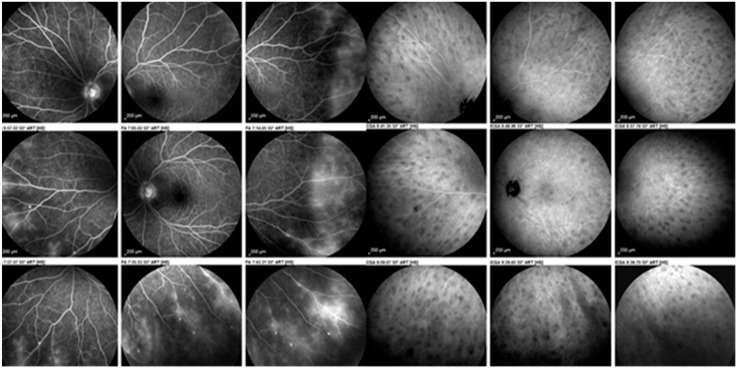
Ocular sarcoidosis, angiographic signs: sarcoidosis chorioretinitis involving both the retina as shown on the nine fluorescein angiographic panorama pictures on the left with a diffuse vasculitis (leaking retinal vessels) as well as the choroid shown on the nine indocyanine green panorama pictures on the right with numerous hypofluorescent dark dots (granulomas).

**Figure 6:**
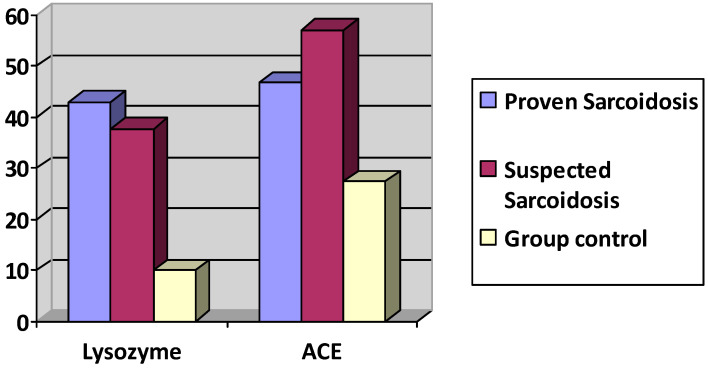
Lysozyme and ACE serum levels in proven ocular sarcoidosis, suspected OS and control group.

**Figure 7:**
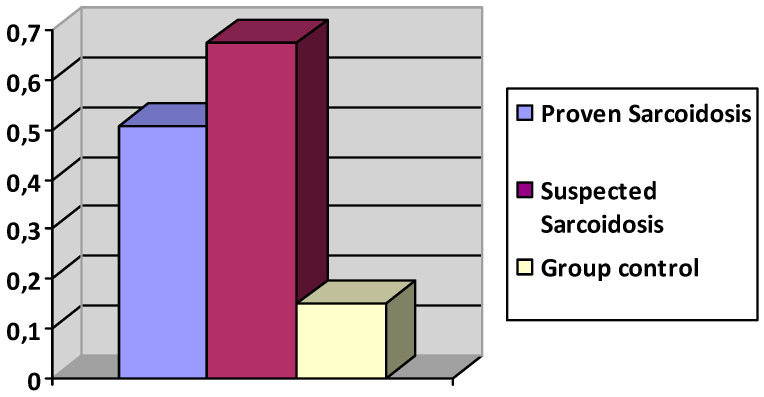
Polyclonal activation ratio in groups with proven, suspected sarcoidosis, and controls.

The second category of sarcoidosis suspect patients corresponded to Levels 3 and 4 of the IWOS criteria when five of the following clinical, angiographic and immunological signs were positive: (1) mutton-fat KPs (large and small); (2) iris nodules at the pupillary margin (Koeppe) or in the iris stroma (Busacca) (Figure 1); (3) trabecular meshwork (TM) nodules and/or tent-shaped peripheral anterior synechiae (PAS); (4) snowballs/string of pearls vitreous opacities; (5) multiple chorioretinal peripheral lesions (active and atrophic) (Figure 2); (6) nodular and/or segmental peri-phlebitis (±candle-wax drippings) (Figure 3) and/or microaneurysm in an inflamed eye **(**Figure 4); (7) optic disc nodule(s)/granuloma(s) and/or solitary choroidal nodule; (8) bilaterality (assessed by clinical examination or investigational tests showing subclinical inflammation such as laser flare photometry (LFP)); (9) negative tuberculin test in a BCG vaccinated patient or having had a positive PPD (or Mantoux) skin test previously; and (10) indocyanine green angiographic signs showing choroiditis or chorioretinitis (Figure 5). Items 1, 2 and 8 were required.

### 2.2. Serum ACE and Lysozyme Analysis

The levels of serum ACE and lysozyme were evaluated.

Serum ACE activity was measured using FAPGG (N-[3-(2-furyl) acryloyl]-L-phenylalanyl-glycylglycine; Sigma-Aldrich) with a Buhlmann kit [12]. Normal values for adults are 20–70 U/I and 29–112 U/I for children under 18 years old.

Serum lysozyme activity was measured by using radial immunodiffusion (Mancini G. et al.) [13], with NANORID™ kits (Binding Site Ltd., Birmingham, UK). Normal values are 9.6–17.1 mg/L for all ages.

The means of serum ACE and lysozyme values in the ocular sarcoidosis groups (proven and suspected) were compared to a control group of non-granulomatous uveitis (pars-planitis and HLA B27 related uveitis) for which these tests had been performed as part of the work-up before the final diagnosis was known. The control groups were chosen based on the availability of each test.

Furthermore, patients were classified into four groups regarding elevation of both tests to compare their proportion: (1) both tests normal; (2) ACE elevated, lysozyme normal; (3) ACE normal, lysozyme elevated; and (4) both tests elevated.

### 2.3. Polyclonal Antibody Activation as a Marker for (Ocular) Sarcoidosis

Compensatory increase of immunoglobulins as a result of decrease of T cell activity in sarcoidosis has been published in many reports [2,3,4,5,6,7]. We showed this and quantified the phenomenon in a previous study and followed the same protocol in the present study [8]. Briefly, we chose to test the exposition of the patients to four human commensal herpesviruses, namely EBV (Epstein-Barr virus), CMV (Cytomegalovirus), HSV (Herpes Simplex virus) and VZV (varicella-zoster virus), by ELISA or Immunofluorescence. In parallel, quantitative serology was performed by complement fixing serologies. For EBV, CMV and HSV, titers ≥ 1/40 were considered elevated and for VZV titers > 1/10 were considered elevated. The polyclonal activation ratio was calculated by dividing the number of elevated serologies by the number of viruses to which the patient had been exposed. For instance, when a patient was exposed to all four viruses and three serologies were elevated by complement fixation, the ratio was 3/4 = 0.75. For this parameter, the mean of ratios in the ocular sarcoidosis groups was compared with a control group of non-granulomatous uveitis (pars planitis and HLA-B27 related uveitis) for whom this test had been performed as part of the work-up before the final diagnosis was known.

## 3. Results

### 3.1. Demographics

Among the 1130 patients seen from 2005 to 2020, 168 patients (14.8%) were diagnosed as possible ocular sarcoidosis patients. Applying the inclusion criteria defined in the previous section, 37 patients (24 female and 13 male patients) could be included (17 patients with IWOS Level 1 and 2 criteria qualifying for proven sarcoidosis and 20 ocular sarcoidosis suspect patients). Mean age was 54.52 ± 23.74. The control group mean age was variable (*n* = 30 mean age was 41 ± 11.3, *n* = 47 mean age was 45.2 ± 17.6 and *n* = 42 mean age was 45.4 ± 17).

### 3.2. Serum ACE and Lysozyme Levels

Mean serum levels of ACE were 49.17± 29 IU/L in the ocular sarcoidosis group versus 27.4 ± 15.34 IU/L (*p* ≤ 0.00018, student’s t test) in the control group (*n* = 30). Mean serum lysozyme levels were 39.92 ± 55.5 mg/L in the ocular sarcoidosis group versus 10.05 ± 5.88 mg/L (*p* ≤ 0.0013) in the control group (*n* = 47) (Figure 6).

The group with proven sarcoidosis (*n* = 17) presented an ACE level of 46.69 ± 23 IU/L and a lysozyme level of 43± 67 mg/L. The group with suspected sarcoidosis (*n* = 20) presented an ACE level of 56.93 ± 32.21 IU/L (*p* = 0.2603 student’s t test comparing to group of proven sarcoidosis) and a lysozyme level of 37.69 ± 47.87 mg/L (*p* = 0.8210 student’s t test, comparing to group of proven sarcoidosis).

The proportion of ACE and lysozyme of patients having both tests elevated was 9/37 (24.3%) (Table 1). The proportion of patients having an elevated ACE serum and a normal level of lysozyme was 2/37 (5.4%), whereas the proportion of patients with a normal level of serum ACE and an elevated level of serum lysozyme was much higher with a proportion of 22/37 (59.4%). The proportion of patients having neither test elevated was 4/37 (10.8%).

### 3.3. Polyclonal Antibody Activation Results

The mean score of polyclonal activation was 0.6 ± 0.33 in the ocular sarcoidosis group versus 0.15 ± 0.2 for the control group (*n* = 42) (*p* ≤ 0.00001), showing a large statistically significant difference of increase of polyclonal activation in the ocular sarcoidosis group.

The group with proven sarcoidosis (*n* = 17) presented a polyclonal activation ratio of 0.508 ± 0.356. The group with suspected sarcoidosis (*n* = 20) presented a polyclonal activation at 0.674 ± 0.28 (Figure 7).

Sensitivity was 70%, specificity was 90.4%. Positive Predictive Value (PPV) was 86.6% and Negative Predictive Value (NPV) was 77.5% (Table 2).

## 4. Discussion

Ocular sarcoidosis has a heterogenous presentation rendering diagnosis sometimes difficult. A combination of clinical signs, investigational and laboratory tests with the highest possible sensitivities and specificities has to be sought to achieve the highest probability of correct diagnosis. At present, paraclinical investigations are far from being satisfactory. Therefore, a combination of multiple tests is performed to increase the accuracy of diagnosis. Recent studies have been published proposing new potentially more specific and sensitive biomarkers for sarcoidosis such as soluble interleukin-2 receptor (sIL-2R) [15] or the elevation of Krebs von den Lungen-6 (KL-6), a human MUC1 mucin protein [16]. Both biomarkers where not included in the 2017 revised IWOS diagnostic criteria as they are not used widely by ophthalmologists or their role is not fully understood in ocular sarcoidosis [17].

On the contrary, high serum lysozyme was newly included as a criterion separate from ACE for the diagnosis of ocular sarcoidosis in the revised IWOS diagnostic criteria in 2017 [17]. Nevertheless, in our daily experience as a reference center of uveitis patients, lysozyme is rarely ordered by general ophthalmologists in the investigation of OS. This may be explained, on the one side, by the fact that many laboratories do not offer the test, as it is complicated to perform and poorly remunerated. On the other side, as it is rarely reported in studies on OS and is not “in the habits” of the ophthalmologist to ask for this test, most centers limit themselves to report ACE levels. It was our feeling that testing for ACE was disappointing while serum lysozyme seemed to be much more rewarding. Indeed, our study showed that lysozyme was much more useful than ACE in OS. Thirty-one of the 37 patients (83%) with proven and/or suspected ocular sarcoidosis were found to have a high level of lysozyme, while this was the case for ACE in only 11 of the 37 patients (29.7%). Especially in patients with proven ocular sarcoidosis, the mean serum level of lysozyme was higher than in the group of suspected ocular disease. In addition, we found that lysozyme was much more sensitive than ACE in the diagnosis of ocular sarcoidosis reaching 83.7% versus 27% for ACE (Table 2). We also proved that both tests have high positive predictive values. The probability that a patient has the disease when lysozyme and ACE are elevated is high, as the percentage is more than 90% (91.9% for lysozyme and 90.9% for ACE). On the contrary, the probability of a patient to be disease-free when lysozyme is negative is 81.8%, much higher than for ACE, reaching only 51.7%. Our results are in accordance with the studies by Kawaguchi et al. [18] and Baarsma et al. [19] who found that sensitivity in OS diagnosis, PPV and NPV were higher with lysozyme than with ACE.

Another limitation of the usefulness of ACE serum levels as diagnostic test for OS is the abolition of ACE serum activity in patients taking angiotensin-converting enzyme inhibitors (ACEIs) to treat systemic hypertension. [20] ACE levels are not similarly affected by all drugs of this category, as patients treated with zofenopril have been reported as having significantly higher ACE levels with respect to those treated with ramipril, enalapril and perindopril, being however sufficient in all cases to disqualify this diagnostic test [20].

Finally, additional investigational tests are certainly welcome. In a previous study, we found that the role of polyclonal antibody activation of four human commensal herpesviruses, as a result of decrease of T cell activity captured in sarcoidosis granulomas, appeared as a useful marker for the diagnosis of ocular sarcoidosis [8]. Indeed, we confirmed in the present study that the ratio of polyclonal activation was significantly increased in sarcoidosis patients, compared to the control group. Sensitivity of polyclonal antibody activation reached 70% while specificity was 90.4%; the probability that a patient has ocular sarcoidosis when polyclonal antibody activity is present amounts to 86.6% (Table 2)**.**

## 5. Conclusions

Ocular sarcoidosis should be considered in the differential diagnosis of any ocular inflammation, especially if granulomatous signs are present, and a laboratory test can be helpful to obtain a more certain diagnosis. Lysozyme seems to be more helpful than ACE in the diagnosis of ocular sarcoidosis. Polyclonal antibody activation to herpes viruses is another useful biomarker. 

## Figures and Tables

**Table 1:** Proportion of ACE and Lysozyme elevation.

Both Tests Elevated	9/37	24.3%
ACE Elevated, Lysozyme Normal	2/37	5.4%
ACE Normal, Lysozyme Elevated	22/37	59.4%
Both Tests Normal	4/37	10.8%

**Table 2:** Predictive values of laboratory tests for the diagnosis of Ocular Sarcoidosis.

Laboratory Test	Sensitivity	Specificity	PPV	NPV
High Serum ACE	0.270	0.966	0.909	0.517
High Serum Lysozyme	0.837	0.900	0.911	0.818
Polyclonal Activation	0.700	0.904	0.866	0.775

## Data Availability

For data availability: contact to corresponding author.

## Abbreviations

ACE	angiotensin converting enzyme
OS	ocular sarcoidosis
IWOS	international workshops on ocular sarcoidosis
BHL	bilateral hilar lymphadenopathy
LFP	laser flare photometry
EBV	epstein-barr virus
CMV	cytomegalovirus
HSV	herpes simplex virus
VZV	varicella-zoster virus
PPV	positive predictive value
NPV	negative predictive value
ACEIs	angiotensin-converting enzyme inhibitors

## References

[1] Herbort C.P., Rao N.A., Mochizuki M., Scientific Committee of the First International Workshop on Ocular Sarcoidosis (IWOS) (2009). International criteria for the diagnosis of ocular sarcoidosis: Results of the first International Workshop On Ocular Sarcoidosis (IWOS). Ocul. Immunol. Inflamm..

[2] James D.G. (1977). Pathobiology of sarcoidosis. Pathobiol. Annu..

[3] James D.G., William W.J. (1982). Immunologgy of sarcoidosis. Am. J. Med..

[4] James D.G. (1986). Ocular sarcoidosis. Ann. N. Y. Acad. Sci..

[5] Daniele R.P., Dauber J.H., Rossman M.D. (1980). Immunologic abnormalities in sarcoisosis. Ann. Int. Med..

[6] Wolska-Goszka L., Cynowska B., Sztaba-Kania M., Jassem E., Slominski J.M. (1992). Evaluation of the activity of angiotensin I convert-ing enzyme (ACE) and humoral immunity in patients with active pulmonary sarcoidosis. Wiad. Lek..

[7] Tannenbaum H.E., Rocklin R., Schur P.H., Sheffer A.L. (1976). Immune function in sarcoidosis. Studies on delayed hypersensitivity, B and T lymphocytes, serum immunoglobulins and serum complement components. Clin. Exp. Immunol..

[8] Berthoud-Kündig J.F., Keller A., Herbort C.P. (1994). Increase in polyclonal immunoglobulins: A possible useful aid in diagnosis of uvei-tis caused by sarcoidosis. Klin. Mon. Augenheilkd..

[9] Ramos-Casals M., Retamozo S., Sisó-Almirall A., Pérez-Alvarez R., Pallarés L., Brito-Zerón P. (2019). Clinically-useful serum biomarkers for diagnosis and prognosis of sarcoidosis. Expert Rev. Clin. Immunol..

[10] Sahin O., Ziaei A., Karaismailoğlu E., Taheri N. (2016). The serum angiotensin converting enzyme and lysozyme levels in patients with ocular involvement of autoimmune and infectious diseases. BMC Ophthalmol..

[11] Febvay C., Kodjikian L., Maucort-Boulch D., Perard L., Iwaz J., Jamilloux Y., Broussolle C., Burillon C., Seve P. (2015). Clinical features and diagnostic evaluation of 83 biopsy-proven sarcoid uveitis cases. Br. J. Ophthalmol..

[12] Beneteau B., Baudin B., Morgant G., Giboudeau J., Baumann F.C. (1986). Automated kinetic assay of angiotensin-converting enzyme in serum. Clin. Chem..

[13] Mancini G., Carbonara A., Heremans J. (1965). Immunochemical quantitation of antigens by single radial immunodiffusion. Immunochemistry.

[14] Varron L., Abad S., Kodjikian L., Seve P. (2011). Uvéites sarcoïdosiques: Actualités diagnostiques et thérapeutiques. La Revue de Médecine Interne.

[15] Schimmelpennink M.C., Quanjel M., Vorselaars A., Wiertz I., Veltkamp M., Van Moorsel C., Grutters J.C. (2020). Value of serum soluble interleukin-2 receptor as a diagnostic and predictive biomarker in sarcoidosis. Expert Rev. Respir. Med..

[16] Ishikawa N., Hattori N., Yokoyama A., Kohno N. (2012). Utility of KL-6/MUC1 in the clinical management of interstitial lung diseases. Respir. Investig..

[17] Mochizuki M., Smith J.R., Takase H., Kaburaki T., Acharya N.R., Rao N.A. (2019). Revised criteria of International Workshop on Ocular Sarcoidosis (IWOS) for the diagnosis of ocular sarcoidosis. Br. J. Ophthalmol..

[18] Kawaguchi T., Hanada A., Horie S., Sugamoto Y., Sugita S., Mochizuki M. (2007). Evaluation of Characteristic Ocular Signs and Systemic Investigations in Ocular Sarcoidosis Patients. Jpn. J. Ophthalmol..

[19] Baarsma G., La Hey E., Glasius E., de Vries J., Kijlstra A. (1987). The Predictive Value of Serum Angiotensin Converting Enzyme and Lysozyme Levels in the Diagnosis of Ocular Sarcoidosis. Am. J. Ophthalmol..

[20] D’Alessandro M., Bergantini L., Perrone A., Cameli P., Cameli M., Prasse A., Plataroti D., Sestini P., Bargagli E. (2020). Serial investigation of Angiotensin-Converting Enzyme in sarcoidosis patients treated with Angiotensin-Converting Enzyme Inhibitor. Eur. J. Intern. Med..

